# Expanding the clinical and molecular spectrum of *TUBB2B* through distinct variants identified across multiple families

**DOI:** 10.1016/j.xhgg.2026.100654

**Published:** 2026-07-17

**Authors:** Shaghayegh T. Beheshti, Angad Jolly, Ahmed K. Saad, Haowei Du, Lauren E. Westerfield, Chloe Munderloh, Divya Kalra, Yifan Wu, Yi Chen, Marie-Claude Gingras, Shalini N. Jhangiani, Sarenur Yilmaz, Maha S. Zaki, Daniel G. Calame, Davut Pehlivan, Richard A. Gibbs, Richard A. Lewis, James R. Lupski, Jennifer E. Posey

**Affiliations:** 1 Department of Molecular and Human Genetics, Baylor College of Medicine, Houston, TX 77030, USA; 2 Department of Obstetrics and Gynecology, Baylor College of Medicine, Houston, TX 77030, USA; 3 Human Genome Sequencing Center, Baylor College of Medicine, Houston, TX 77030, USA; 4 Istanbul Medeniyet University, Istanbul 34000, Turkey; 5 Clinical Genetics Department, Human Genetics and Genome Research Institute, National Research Centre, Cairo 12622, Egypt; 6 Section of Pediatric Neurology and Developmental Neuroscience, Department of Pediatrics, Baylor College of Medicine, Houston, TX 77030, USA; 7 Department of Ophthalmology, Cullen Eye Institute, Baylor College of Medicine, Houston, TX 77030, USA; 8 Department of Pediatrics, Baylor College of Medicine, Houston, TX 77030, USA; 9 Texas Children’s Hospital, Houston, TX 77030, USA; 10 Departments of Pediatrics and Medicine, Vagelos College of Physicians & Surgeons, Columbia University Irving Medical Center, New York, NY 10032, USA

**Keywords:** TUBB2B, tubulinopathy, neuronal migration disorder, corpus callosum agenesis, septo-optic dysplasia, panhypopituitarism, phenotypic heterogeneity, tubulin polyamination, biallelic TUBB2B variants, dual molecular diagnosis

## Abstract

*TUBB2B* encodes a β-tubulin isotype essential for neuronal proliferation, migration, and organization during brain development. Pathogenic heterozygous variants in *TUBB2B* are associated with neurodevelopmental disorders including polymicrogyria and corpus callosum abnormalities. However, the phenotypic spectrum remains heterogeneous, most likely reflecting variant-specific effects on microtubule formation and stability. Homozygous *TUBB2B* variants are exceedingly rare, with one family reported to date. We describe five individuals in four families with rare *TUBB2B* variants. Four variants are described, including a previously reported *de novo* missense variant, c.292G>A (GenBank: NM_178012.5) (p.Gly98Arg), with potential phenotypic expansion including panhypopituitarism; a previously reported *de novo* missense variant, c.605T>C (GenBank: NM_178012.5) (p.Ile202Thr), showing interindividual heterogeneity; a *de novo* missense variant, c.43C>A (GenBank: NM_178012.5) (p.Gln15Lys) at a polyamination site critical for microtubule stability; and a homozygous missense variant within a region of absence of heterozygosity in two siblings from consanguineous parents, c.145G>A (GenBank: NM_178012.5) (p.Val49Ile). Both individuals also carry a pathogenic homozygous truncating *ALKBH8* variant c.1675del (GenBank: NM_138775.3) (p.Arg559Alafs∗56), representing a potential dual molecular diagnosis driving clinical features reflective of contributions from both genes. These reports expand the clinical spectrum of *TUBB2B*-related tubulinopathies, illustrate phenotypic heterogeneity, and provide insights into disease mechanisms including effects at polyamination sites and rare recessive inheritance, underscoring the need for nuanced genotype-phenotype interpretation in diagnostic and counseling contexts.

## Teaser

Four *TUBB2B* variants across five individuals reveal unexpected phenotypes including panhypopituitarism, disruption of a polyamination site critical for microtubule stability, and a dual diagnosis with *ALKBH8*. These findings add critical nuance to the heterogeneous tubulinopathy spectrum and underscore the importance of careful genotype-phenotype interpretation.

## Introduction

Brain development requires tightly regulated neuronal proliferation, migration, and differentiation supported by microtubules (MTs) comprising αβ-tubulin heterodimers forming polar protofilaments.^1^ Disruption of tubulin structure or function causes tubulinopathies.^1^

Variants in 12/19 human α-, β-, and γ-tubulin genes (*TUBA1A* [MIM: 602529], *TUBA4A* [MIM: 191110], *TUBA8* [MIM: 605742], *TUBB1* [MIM: 612901], *TUBB2A* [MIM: 615101], *TUBB2B* [MIM: 612850], *TUBB3* [MIM: 602661], *TUBB* [MIM: 191130], *TUBB4A* [MIM: 602662], *TUBB8* [MIM: 616768], *TUBGCP2* [MIM: 617817], and *TUBG1* [MIM: 191135]) cause rare central nervous system disorders^1^^,^^2^ characterized by neuronal migration defects, corpus callosum agenesis, basal ganglia abnormalities, and hindbrain malformations.^1^ Damaging variants produce distinct phenotypes through variable effects on MT function^3^: disrupting heterodimer supply, GTP binding, protofilament interactions, or MT-microtubule-associated protein (MAP) interactions.^4^

*TUBB2B* is brain-expressed and loss-of-function (LoF) intolerant (gnomAD v.4.1 probability of loss-of-function intolerance [pLI] = 1).^1^ Heterozygous missense variants in *TUBB2B* cause complex cortical dysplasia with other brain malformations (CDCBM7 [MIM: 610031]) via LoF, dominant-negative, and gain-of-function mechanisms. Phenotypic heterogeneity is illustrated by a family with the *TUBB2B* variant, c.530A>T (GenBank: NM_178012.5) (p.Asp177Val): the mother had childhood language delay, the son had mild neurodevelopmental delay with tubulinopathy-consistent MRI findings, and a fetus had microcephaly and major brain anomalies.^5^ A homozygous *TUBB2B* variant, c.1169G>A (GenBank: NM_178012.5) (p.Arg390Gln), was reported in three individuals with Uner Tan syndrome from a consanguineous Turkish family.^6^ Functional studies showed preserved heterodimer formation but reduced MT stability, demonstrating variable *TUBB2B* variant effects.^6^

We report four unrelated families with *TUBB2B* variants. Three have heterozygous missense variants: the known c.292G>A (GenBank: NM_178012.5) (p.Gly98Arg), now associated with panhypopituitarism, suggesting phenotypic expansion; the recurrent c.605T>C (GenBank: NM_178012.5) (p.Ile202Thr) variant at a predicted critical residue illustrating variant-level phenotypic heterogeneity; and c.43C>A (GenBank: NM_178012.5) (p.Gln15Lys) at a polyamination site important for MT stability. We also describe a consanguineous Egyptian family previously reported with a homozygous *ALKBH8* (MIM: 613306) variant,^7^ c.1675del (GenBank: NM_138775.3) (p.Arg559Alafs∗56), with a homozygous *TUBB2B* variant, c.145G>A (GenBank: NM_178012.5) (p.Val49Ile), identified in affected siblings. We present clinical and molecular findings expanding the genotypic and phenotypic spectrum of *TUBB2B*-related disorders (Table 1).

**Table 1:** Clinical features of individuals with *TUBB2B* variants annotated using Human Phenotype Ontology terms

Phenotype category	Phenotype	HPO ID	Individual 1 (BAB11820)	Individual 2 (NC33054)	Individual 3 (BAB9222)	Individual 4 (BAB13277)	Individual 5 (BAB13280)
Demographics	age at last report	–	4 years	9 years, 4 months	33 months	3 years	14 years
	sex	–	male	male	male	male	female
	ancestry	–	European	Algerian	Turkish	Egyptian	Egyptian
	pregnancy and delivery	–	full term, no complications	full term, no complications	full term, no complications	full term, no complications	full term, no complications
Growth parameters	height	–	1.26 SD (at birth)	0.3 SD (at birth)	−1.12 SD (at birth)	−2.7 SD (3 years old)	−5.3 SD (14 years old)
	weight	–	−0.54 SD (at birth)	−0.9 SD (at birth)	−0.35 SD (at birth)	−2.1 SD (3 years old)	−2.6 SD (14 years old)
	head circumference (HC)	–	−2.02 SD (6 days)	−1.3 SD (at birth)	−0.42 SD (at birth)	−0.9 SD (3 years old)	−2.4 SD (14 years old)
Neurological findings	corpus callosum atrophy	HP:0007371	yes (agenesis)	yes (hypogenesis)	yes (agenesis)	yes (agenesis)	yes (agenesis)
	ventriculomegaly	HP:0002119	–	yes	–	yes	yes
	delayed CNS myelination	HP:0002188	–	–	–	yes	yes
	cerebral hypoplasia	HP:0006872	yes	–	–	–	–
	cerebellar hypoplasia	HP:0001321	–	yes	–	yes	yes
	cortical dysplasia	HP:0002539	no	yes (abnormal gyration, thick cortex)	no	no	no
	thinning of brain stem	–	–	yes (mainly pons)	–	–	–
Seizures/epilepsy	seizure	HP:0001250	yes	no	yes	no	no
Developmental	intellectual disability	HP:0001249	–	yes	–	yes	yes
	motor delay	HP:0001270	yes	yes	yes	yes	yes
	speech delay	HP:0000750	yes	yes	yes	yes	yes
	global developmental delay	HP:0001263	–	yes	–	yes	yes
Behavioral features	autism	HP:0000717	–	–	yes	–	yes
	happy demeanor	HP:0040082	–	–	yes	–	–
	attention-deficit hyperactivity disorder	HP:0007018	–	–	–	yes	–
	mood swings	–	–	yes	–	–	–
	hyperactivity	HP:0000752		yes	–	–	yes
Vision/ophthalmological findings	optic nerve hypoplasia	HP:0000609	yes	–	–	–	–
	astigmatism	HP:0000483	yes	–	–	–	–
	nystagmus	HP:0000639	yes	–	–	–	–
Craniofacial findings	dysmorphic facial features	HP:0001999	–	yes	yes	yes	yes
Other neurological features	microcephaly	HP:0000252	yes	yes	–	–	–
	cerebral palsy	HP:0100021	yes	–	–	–	–
	hypotonia	HP:0001252	–	yes	yes	yes	yes
	hyporeflexia	HP:0001265	–	–	–	yes	yes
Genitourinary findings	bilateral cryptorchidism	HP:0000028	–	–	–	yes	–
Skin findings	hair hypopigmentation	HP:0005599	–	–	yes	–	–
Other medical features	panhypopituitarism	HP:0000871	yes	–	–	–	–
	delayed puberty	HP:0000823	–	–	–	–	yes
Studies performed	–	–	prior clinical lab testing positive for the *TUBB2B* variant	normal karyotyping, metabolic workup, fundus examination, ABR, EMG, and NCV	blood NH_3_, plasma aa, urine OA, tandem MS, and MR spectroscopy were negative; hearing, ECHO, and EMG were normal; karyotype was 46,XY; Angelman testing (FISH for SNRPN, methylation) was negative	–	–
*TUBB2B* (GenBank: NM_178012.5)	–	–	c.292G>A (p.Gly98Arg)	c.605T>C p.(Ile202Thr)	c.43C>A (p.Gln15Lys)	c.145G>A (p.Val49Ile)	c.145G>A (p.Val49Ile)

This table summarizes phenotypic findings in five affected individuals across four families carrying rare missense variants in the *TUBB2B* gene. Phenotypes are categorized by clinical system and annotated using Human Phenotype Ontology (HPO) identifiers. Growth parameters are reported as standard deviation (SD) scores where available. Yes indicates that the phenotype was documented in the clinical record. No indicates the feature was absent, and cells with an en dash (–) indicate the feature was not reported. Individuals 4 and 5 are siblings with a homozygous variant in *TUBB2B*, while individuals 1, 2, and 3 each harbor *de novo* heterozygous variants. ABR, auditory brainstem response; EMG, electromyography; NCV, nerve conduction velocity; NH_3_, blood ammonia; aa, plasma amino acids; OA, urine organic acids; tandem MS, tandem mass spectrometry; MR spectroscopy, magnetic resonance spectroscopy; ECHO, echocardiogram; FISH, fluorescence *in situ* hybridization; SNRPN, small nuclear ribonucleoprotein polypeptide.

## Material and methods

Study procedures complied with the ethical standards of the responsible committee on human experimentation, and written informed consent was obtained for all participants. Families 1, 3, and 4 were enrolled under Baylor College of Medicine (BCM) institutional review board (IRB) protocol H-29697 through the Baylor-Hopkins Center for Mendelian Genomics^8^ and family 2 under a protocol at the National Research Center (Cairo, Egypt). DNA was extracted from blood and trio exome sequencing (ES) performed using established protocols.^9^ Individual 2 underwent commercial ES. Exome capture for Families 1, 3, and 4 used the BCM Human Genome Sequencing Center design (52-Mb, Roche NimbleGen, RRID: nif-0000-31466), with Illumina HiSeq (∼100× coverage). Reads were aligned to hg19 using Mercury,^10^ and variants called with ATLAS2 (individual 3) or xATLAS^11^ (individuals 1 and 4) and annotated as described.^12^

Absence of heterozygosity (AOH), a surrogate for runs of homozygosity, was detected from unphased exome data using BafCalculator (https://github.com/BCM-Lupskilab/BafCalculator).^13^ B allele frequency was calculated as the alternate-to-total read proportion at variant sites. AOH regions were defined using a circular binary segmentation algorithm.^14^

Exomes were analyzed for rare, damaging variants under autosomal and X-linked dominant and recessive models. Variants with minor-allele frequency < 0.1% in gnomAD v.2/v.4 were prioritized using *in silico* scores: Combined Annotation Dependent Depletion (CADD),^15^ AlphaMissense,^16^ and REVEL.^15^ Copy-number variants (CNVs) were assessed using XHMM^17^ and HMZDelFinder.^18^

**Figure 1:**
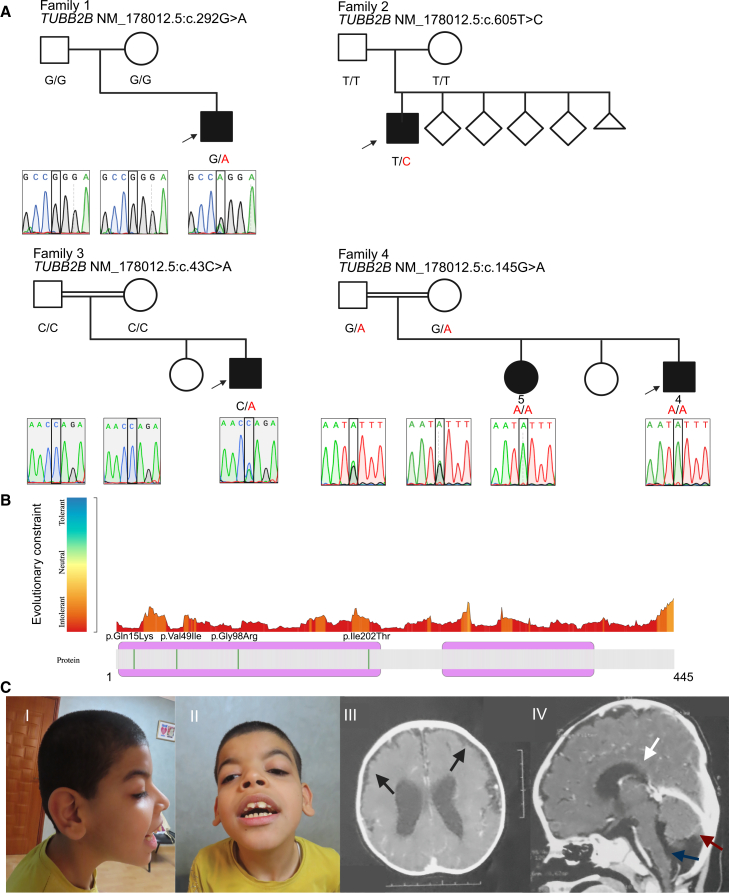
Identification and characterization of *TUBB2B* variants in four families with affected individuals and associated neuroimaging and clinical features of NC33054 (A) Pedigrees for all families and Sanger sequencing chromatograms for three families with rare *TUBB2B* missense variants. Individual 1 (BAB11820) carries a *de novo* heterozygous c.292G>A (GenBank: NM_178012.5) (p.Gly98Arg) variant. Individual 2 (NC33054) carries a *de novo* heterozygous c.605T>C (GenBank: NM_178012.5) (p.Ile202Thr) variant. Individual 3 (BAB9222) carries a *de novo* heterozygous c.43C>A (GenBank: NM_178012.5) (p.Gln15Lys) variant. Family 4 includes two affected siblings, individuals 4 (BAB13277) and 5 (BAB13280), with a homozygous c.145G>A (GenBank: NM_178012.5) (p.Val49Ile) variant, inherited from heterozygous carrier parents. Sanger sequencing confirmed the variants and their segregation within each family. (B) Intolerance landscape of the TUBB2B protein generated using MetaDome v.1.0.1. Regions of the protein are color coded based on evolutionary constraint, ranging from tolerant (blue) to intolerant (red). The four variants identified in this study, p.Gln15Lys, p.Val49Ile, p.Gly98Arg, and p.Ile202Thr, are indicated by vertical green lines and are located in highly intolerant regions of the protein, supporting their damagingness. (C) Clinical photographs of individual 2 showing (I and II) characteristic facial dysmorphism, including a long face, narrow forehead, arched eyebrows, bilateral ptosis, eyelid puffiness, upturned nose, short philtrum, bow-shaped lips with broad, prominent upper incisors, and large, low-set ears with prominent lobules. (III) Axial brain CT demonstrating abnormal gyral patterns (black arrows), cortical thickening, and mild dilation of the lateral ventricles. (IV) Brain CT showing hypogenesis of the corpus callosum (white arrow), a small cerebellar vermis (red arrow), and thinning of the brainstem (blue arrow), most prominently involving the pons. Created with BioRender.com.

Variants were confirmed by PCR and Sanger sequencing (Figure 1A; Table S1).

## Results

Five individuals from four families are described.

### Individual 1

The proband is a 4-year-old European male born at term. Birth weight was 3.23 kg (Z = −0.54) and length 53.3 cm (Z = +1.26). Head circumference (HC) at 6 days was 31.8 cm (Z = −2.02).

Head ultrasound at 2 weeks showed corpus callosum agenesis and ventriculomegaly confirmed by CT and MRI at 2 months. Optic nerves were ∼30% of expected size, initially attributed to microcephaly. By age 2, panhypopituitarism with central hypothyroidism and adrenal insufficiency was diagnosed. The triad of corpus callosum agenesis, panhypopituitarism, and optic nerve hypoplasia established a diagnosis of septo-optic dysplasia (SOD). Additional features included global developmental delay requiring physical and speech therapy, cortical visual impairment, astigmatism, acquired nystagmus, and seizures.

At 2.5 years, weight was 14.77 kg (Z = +0.54), height 91.5 cm (Z = −0.61), and HC 42.2 cm (Z = −4.12). Brain MRI showed cerebral atrophy. Exam revealed hypotonia and poor visual tracking.

Clinical trio-exome reanalysis identified a *de novo* heterozygous missense variant in *TUBB2B*, c.292G>A (GenBank: NM_178012.5) (p.Gly98Arg), absent from gnomAD v.4 and internal databases, predicted deleterious by *in silico* models (Table 2), and mapped to a constrained region (Figure 1B). Sanger sequencing confirmed *de novo* status (Figure 1A).

**Table 2:** Zygosity and predicted damaging scores for missense variants identified in affected individuals

Individual ID	Gene	Transcript	Variant (cDNA:protein)	Zygosity	CADD	REVEL	Alpha missense	gnomAD v.4	ACMG variant classification criteria
Individual 1 (BAB11820)	*TUBB2B*	NM_178012.5	c.292G>A (p.Gly98Arg)	heterozygous (*de novo*)	25.3	0.836	0.99	0	PS2_VeryStrong^a^PP3_ModeratePM2_SupportingPP2_SupportingPP5_Supportingpathogenic
Individual 2 (NC33054)	*TUBB2B*	NM_178012.5	c.605T>C (p.Ile202Thr)	heterozygous (*de novo*)	24.7	0.96	0.96	0	PS2_VeryStrong^b^PP3_StrongPM2_SupportingPP2_Supportingpathogenic
Individual 3 (BAB9222)	*TUBB2B*	NM_178012.5	c.43C>A (p.Gln15Lys)	heterozygous (*de novo*)	28.5	0.589	0.99	0	PS2_StrongPM2_SupportingPP2_Supportinglikely pathogenic
Individuals 4 (BAB13277) and 5 (BAB13280)	*TUBB2B*	NM_178012.5	c.145G>A (p.Val49Ile)	homozygous	25	0.52	0.57	0	PP1_ModeratePM2_SupportingPP2_SupportingPM5_SupportingVUS
	*ALKBH8*	NM_138775.3	c.1675delC (p.Arg559Alafs∗56)	homozygous	–	–	–	0	PVS1, PM2, PP1pathogenic

This table summarizes the zygosity and *in silico* damaging predictions for four missense variants in *TUBB2B* (transcript GenBank: NM_178012.5) identified in five affected individuals and a previously identified variant in *ALKBH8* (transcript GenBank: NM_138775.3) in two affected individuals. Scores include CADD (v.1.6), where values above 20 suggest deleteriousness; REVEL and AlphaMissense, both of which range from 0 to 1 with higher scores indicating greater damaging potential. All variants are absent from the gnomAD v.4 database. ACMG criteria are shown for each variant, as entered in ClinVar. VUS, variant of uncertain significance.

a Using published sequence variant interpretation guidelines and pathogenic criteria, as well as guidelines proposed by the ClinGen Sequence Variant Interpretation Working Group (https://clinicalgenome.org/working-groups/sequence-variant-interpretation/), we apply the PS2_VeryStrong criterion based on the present report and prior report of 2 individuals with *de novo* c.292G>A (GenBank: NM_178012.5) variants.

b Using published sequence variant interpretation guidelines and pathogenic criteria, as well as guidelines proposed by the ClinGen Sequence Variant Interpretation Working Group (https://clinicalgenome.org/working-groups/sequence-variant-interpretation/), we apply the PS2_VeryStrong criterion based on the present report and prior report of 3 individuals with *de novo* c.605T>C (GenBank: NM_178012.5) variants.

### Individual 2

The proband is a 9-year-4-month-old Algerian male born at term. Birth weight was 3.0 kg (Z = −0.9), length 51 cm (Z = +0.3), and HC 33 cm (Z = −1.3). He has four healthy siblings and no similarly affected relatives (Figure 1A).

Clinical evaluation showed global developmental delay. He sat independently at 1 year, 8 months, walked with support at 5 years, and took a few independent steps at 8 years. Expressive language was limited to three words. He understood simple commands, identified some body parts, and had no bowel/bladder control. Behavioral features included hyperactivity and mood swings. No seizures were observed.

At examination, weight was 25 kg (Z = −1.1), height 135 cm (Z = +0.08), and HC 47.5 cm (Z = −4). Dysmorphic features included a long face, narrow forehead, arched eyebrows, bilateral ptosis, periorbital fullness, upturned nose, short philtrum, bow-shaped lips with broad prominent upper incisors, and large low-set ears with prominent lobules (Figure 1C).

Neurologic assessment revealed drooling, hypotonia, and preserved deep tendon reflexes. Brain CT showed abnormal gyration, thickened cortex, mildly enlarged lateral ventricles, corpus callosum hypogenesis, small cerebellar vermis, and brainstem thinning most pronounced at the pons (Figure 1C).

Clinical exome analysis following negative diagnostic evaluation (Table 1) identified a *de novo* heterozygous missense variant in TUBB2B c.605T>C (GenBank: NM_178012.5) (p.Ile202Thr). Absent from gnomAD v.4, the variant affects a conserved residue (Figure 1B). *In silico* tools predicted deleteriousness (Table 2). Parental segregation confirmed *de novo* status (Figure 1A).

### Individual 3

The proband is a 33-month-old Turkish male born at term to first-cousin consanguineous parents. Birth weight was 3.34 kg (Z = −0.35), length 47 cm (Z = −1.12), and HC 35 cm (Z = −0.42). There was one healthy female sibling (Figure 1A).

Clinical evaluation showed global developmental delay with independent walking at 23 months. He had autistic features, a happy demeanor, and seizures. EEG revealed bilateral occipitotemporal abnormalities with spike-wave discharges. Brain MRI showed diffuse corpus callosum hypoplasia. Mild dysmorphic features included downslanting palpebral fissures, a bulbous nasal tip, tented upper lip, and white-yellow hair depigmentation.

Research exome analysis identified a *de novo* heterozygous missense variant in TUBB2B, c.43C>A (GenBank: NM_178012.5) (p.Gln15Lys), absent from gnomAD v.4. It affects a conserved polyamination site important for MT stability (Figure 1B). *In silico* tools predicted deleteriousness (Table 2). Sanger sequencing confirmed *de novo* status (Figure 1A).

### Individual 4

Previously described by Saad et al.,^7^ the proband (BAB13277) is a 3-year-old Egyptian male born at term to first-cousin consanguineous parents. He has one similarly affected sister (age 14) and one healthy sister (Figure 1A).

Features consistent with homozygous *ALKBH8* variant c.1675del (GenBank: NM_138775.3) (p.Arg559Alafs∗56) included global developmental delay, attention and speech deficits, hypotonia, and distinctive craniofacial features (Table 1). He also had bilateral undescended testes and an IQ of 51 (Stanford-Binet). At 3 years, weight was 11.3 kg (Z = −2.1), height 83 cm (Z = −2.7), and HC 49 cm (Z = −0.9). He had hyporeflexia and brain MRI findings not previously linked to *ALKBH8*: mild ventriculomegaly, volume loss, cerebellar hypoplasia, corpus callosum thinning, and abnormal myelination (Figure 1C).

The affected sister (individual 5) had global developmental and speech delay, hypotonia, mild hyperactivity, autistic traits, and dysmorphic features (Table 1). Her IQ was measured at 42 (WISC), with limited speech progress despite intervention. At 14 years, her weight was 28 kg (Z = −2.6), height 128 cm (Z = −5.3), and HC 51 cm (Z = −2.4). She showed no sexual maturation. Brain MRI revealed mild-moderate cerebellar hypoplasia and infratentorial volume loss more pronounced than her brother, with ventriculomegaly, corpus callosum thinning, and abnormal myelination. Neither sibling had seizures, unlike some reported *ALKBH8* individuals. Birth HC was unavailable.

Prior ES identified a homozygous insertion or deletion (indel) variant in *ALKBH8*, c.1675del (GenBank: NM_138775.3) (p.Arg559Alafs∗56), causing a frameshift terminating in the last exon in both affected siblings. Reanalysis revealed an additional homozygous *TUBB2B* variant, c.145G>A (GenBank: NM_178012.5) (p.Val49Ile), resulting in a missense substitution. This variant was absent from control databases, predicted damaging (Table 2), and located in a constrained region (Figure 1B).

**Figure 2:**
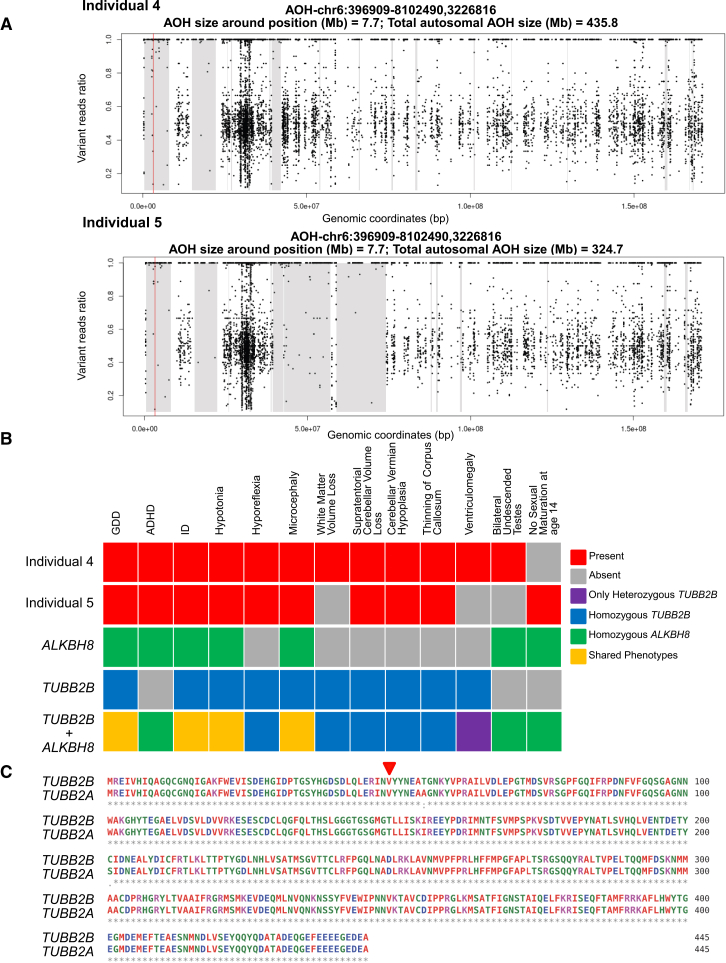
AOH plots and genomic and phenotypic evidence supporting dual molecular diagnosis in siblings with homozygous *TUBB2B* and *ALKBH8* variants (A) Absence of heterozygosity (AOH) plots for the proband (individual 4, top) and the older sister (individual 5, bottom), generated from unphased exome sequencing data using BafCalculator, spanning from base pair 396,909 to 8,102,490 (AOH-chr6:396909–8102490). Both individuals exhibit a 7.7 Mb region of AOH encompassing the *TUBB2B* locus on chromosome 6. Within this region, a red vertical line marks the genomic position chr6:3,226,816 at the variant location. Total autosomal AOH was 435.8 Mb in the proband and 324.2 Mb in the older sister, consistent with parental consanguinity. (B) Phenotypic comparison matrix highlighting the contribution of dual molecular diagnoses in the affected siblings (BAB13277 and BAB13280) with homozygous variants in *TUBB2B* and *ALKBH8*. Red tiles indicate observed phenotypes in each individual. Reference phenotype profiles were compiled from one published individual with homozygous *TUBB2B* (blue)^6^ and individuals with homozygous *ALKBH8* (green)^28^ and heterozygous *TUBB2B* (purple).^4^ Shared features between the two gene-associated phenotypes are shown in yellow. This comparative analysis reveals that several neurological and brain imaging features previously attributed to *ALKBH8* (e.g., cerebellar hypoplasia and corpus callosum thinning) are more consistent with the known spectrum of *TUBB2B*-associated disease, supporting a dual diagnosis rather than a phenotypic expansion. (C) Protein sequence alignment of TUBB2B and TUBB2A using CLUSTAL O (1.2.4) shows 90% sequence homology. The valine at position 49, indicated by the red arrow, is conserved in both proteins. Created with BioRender.com.

To assess consanguinity, unphased ES data were analyzed using BafCalculator. Large AOH intervals included the *TUBB2B* variant within a 7.7-Mb region on chromosome 6 in both siblings (Figure 2A) and the *ALKBH8* variant within a 10.6-Mb region in the proband (individual 4), with a 10.8-Mb region in the affected sister (individual 5) on chromosome 11. Genome-wide AOH totaled 436.4 in individual 4 and 324.7 Mb in individual 5.These findings, combined with known *TUBB2B* phenotypes, raised the possibility that this variant might represent a second molecular diagnosis consistent with features observed in both affected siblings that were not recognized features of *ALKBH8*-associated conditions (Figure 2B). Sanger sequencing confirmed the variant as homozygous in the affected siblings and heterozygous in both parents (Figure 1A).

## Discussion

We report five individuals from four unrelated families with damaging *TUBB2B* variants, expanding the allelic and phenotypic spectrum of TUBB2B tubulinopathies. MT stability and plasticity are crucial for neuronal structure and function.

Individual 1’s TUBB2B variant is predicted to disrupt tubulin folding and was previously reported in two unrelated heterozygous individuals. One, a 10-year-old female with a *de novo* variant, had microcephaly, optic atrophy, seizures, scoliosis, polymicrogyria-like cortex, psychomotor delay, corpus callosum agenesis, ventriculomegaly, basal ganglia dysplasia, and mild cerebellar and brainstem hypoplasia.^19^ The second was a fetus with severe lissencephaly including agyria, corpus callosum agenesis, and a normal cerebellum.^20^ This individual overlaps phenotypically with previously reported individuals, including optic nerve hypoplasia, corpus callosum agenesis, seizures, microcephaly, and developmental delay. These reports demonstrate the phenotypic variability among individuals with the same *TUBB2B* variant. We also report panhypopituitarism in individual 1. Corpus callosum agenesis and panhypopituitarism led to clinical confirmation of SOD despite initial attribution of optic nerve hypoplasia to microcephaly, highlighting challenges in early ascertainment. Given *TUBB2B*’s role in early development, this may expand *TUBB2B*’s phenotypic spectrum as pituitary hormone deficiencies may arise when hypothalamic-pituitary structures are affected.^21^ Central endocrine involvement should be considered for *TUBB2B-*related diseases. A rare maternally inherited *PAX2* variant, c.584G>A (GenBank: NM_000278.5) (p.Arg195His), associated with papillorenal syndrome (PAPRS [MIM: 120330]) was detected in individual 1. The proband and mother lack renal findings, consistent with variable expressivity. The mother, otherwise healthy, was previously noted to have mild optic nerve hypoplasia.

In individual 2, we report a TUBB2B variant, p.Ile202Thr, previously reported in three individuals,^22^^,^^23^ enabling phenotype comparison. Shared features include developmental delay/intellectual disability, hypotonia, corpus callosum hypogenesis/thinning, and cerebellar vermis hypoplasia (3/3 each). Cortical dysgyria/polymicrogyria was noted in 2/3 published individuals and in individual 2, while microcephaly was documented in 2/3 previously and confirmed in our proband (−4 SD). Facial dysmorphism (bilateral ptosis, arched eyebrows, and bow-shaped lips) was present in 2/3 individuals and in our proband. Hyperactivity and mood lability were unique to our proband. One reported individual had congenital fibrosis of the extraocular muscles (CFEOM), whereas our proband had bilateral ptosis without ophthalmoplegia, suggesting milder cranial nerve dysinnervation. This demonstrates phenotypic heterogeneity of a recurrent tubulin variant, likely reflecting genetic, epigenetic, or environmental modifiers.

Docking analysis by Mancini et al. demonstrated that Ile202 stabilizes the hydrophobic core of the β-tubulin GTP-binding pocket required for GTP binding. Although Ile202 does not directly contact the GTP, its non-polar side chain maintains pocket integrity.^22^ Threonine substitution is predicted to disrupt this environment, reduce GTP binding affinity, and impair MT polymerization.

Polyamination supports MT stability during brain development, and its disruption can impair MT dynamics and contribute to developmental and neurodegenerative disorders.^24^ The TUBB2B variant in individual 3 affects a conserved polyamination site and has not been previously linked to disease. This variant was reported in one individual with corpus callosum agenesis, ventriculomegaly, speech delay, and mild intellectual disability with a likely pathogenic variant in *MED12L*. Given the variability of *MED12L-*associated phenotypes, dual diagnosis is possible, though *de novo* status of the *TUBB2B* variant was not confirmed.^25^

Individual 3 shows a milder phenotype with corpus callosum hypoplasia, seen in other *TUBB2B* individuals, suggesting subtle effects on MT stability rather than assembly. Both increased and decreased MT stability impair neuronal plasticity: hyperstabilization preserves axons but limits synaptic remodeling and may contribute to neuropsychiatric features. Disrupted tubulin polyamination may therefore reduce MT flexibility and neuronal adaptability without major malformations.

Multilocus pathogenic variation (MPV), for which pathogenic variants at distinct loci yield blended phenotypes, occurs in 3.2%–7.2% of genetically diagnosed individuals^26^ and is enriched in populations with increased homozygosity due to increased AOH burden.^13^ Such overlap can mimic phenotypic expansion at a single gene.^27^ In individual 4, brain abnormalities may reflect a blended *TUBB2B/ALKBH8* phenotype, as similar features have not been observed in other *ALKBH8* individuals.^28^ Exome reanalysis identified a homozygous *TUBB2B* variant in both affected siblings, inherited from heterozygous unaffected parents. Although most *TUBB2B* variants are associated with dominant disease, one homozygous p.Arg390Gln variant has been reported in a Turkish family with three individuals with Uner Tan syndrome.^6^ Affected family members had severe cerebellar hypoplasia manifested by hand walking.^6^ This family shares features with our individual, including global developmental delay, intellectual disability, hyporeflexia, cerebellar hypoplasia, supratentorial cerebellar volume loss, and hypoplasia of the corpus callosum. Some features previously attributed to phenotypic expansion of *ALKBH8* are now better explained by the *TUBB2B* variant in this family (Figure 2B). Subtle MRI abnormalities may occur in heterozygous tubulin variant carriers in the absence of overt clinical features; thus, parental heterozygosity raises the possibility of subclinical neurologic findings.^29^

To assess variant impact, we examined analogous variants in other tubulin genes. TUBB2A, a neuron-specific β-tubulin isotype with 90% homology to TUBB2B, is also associated with tubulinopathies (Figure 2C), although TUBB2B shows higher developmental expression. Two unrelated individuals with *de novo TUBB2A* variants at the same residue, c.145G>A (GenBank: NM_001069.3) (p.Val49Met) and c.146T>G (GenBank: NM_001069.3) (p.Val49Gly), showed overlapping features with individual 4, including microcephaly (1/2), dysmorphic features (1/2), intellectual disability (2/2), dysgyria (1/2), corpus callosum thinning (1/2), and a small cerebellum (1/2), supporting damagingness at this position.^30^

The TUBB2B p.Val49Ile variant has a lower AlphaMissense score (0.575) than the TUBB2A p.Val49Gly and p.Val49Met variants (0.959 and 0.993), suggestive of a milder functional effect and consistent with its presence in apparently unaffected heterozygous parents. 3D structural modeling using mCSM-PPI2 at the *TUBB2B*-*TUBA1B* interface (based on cryoelectron microscopy [cryo-EM] structure 6E7C) revealed no significant change in protein-protein interaction for this variant. Functional studies will be essential to fully characterize this variant’s effect on MT stability.

We report four *TUBB2B* variants in five individuals: a *de novo* variant at a known residue, linked to panhypopituitarism; a recurrent *de novo* variant demonstrating phenotypic heterogeneity; a *de novo* heterozygous variant at a polyamination site crucial for MT stability; and a homozygous missense variant in two siblings, representing potential MPV with blended *ALKBH8/TUBB2B* features. Given variable effects of *TUBB2B* variants on MT dynamics and neuronal development, functional studies are needed to define impacts on tubulin structure and function.

## Data and code availability

All *TUBB2B* variants have been submitted to ClinVar (ClinVar: SCV006308805, SCV006308749, and SCV006308014). For subjects who have provided written informed consent for sharing of their genomic data in controlled access databases, these data will be deposited in AnVIL and/or dbGaP under dbGaP: phs000711.v5.p1 and phs003047.v3.p2. Access to these data may be granted to qualified researchers who meet the criteria for access to confidential human subject data, subject to approval by the appropriate IRB and data access committee at BCM.

## Acknowledgments

This study was supported in part by the GREGoR Consortium under NIH/NHGRI grant U01HG011758 as well as by the Caroline Wiess Law Award at 10.13039/100000990BCM and through institutional support from the 10.13039/100014434Columbia University Irving Medical Center. ES was facilitated by resources at the Human Genome Sequencing Center at BCM. Additional support was provided through the Genetics & Genomics Training Program (T32GM139534) funded by the 10.13039/100000057National Institute of General Medical Sciences. The data for individual 3 were obtained while S.Y. was affiliated with Istanbul Medeniyet University. We thank the study participants and their families for their participation in this research.

## Declaration of interests

J.E.P. serves on the scientific advisory board of MaddieBio. D.P. provides consulting services for Ionis Pharmaceuticals, M2DS Therapeutics, and Acadia Pharmaceuticals. D.K.’s immediate family member is employed at Oxford Nanopore Technologies. The Department of Molecular and Human Genetics at BCM receives revenue from clinical genetic testing completed at Baylor Genetics Laboratory.

## Declaration of generative AI and AI-assisted technologies in the writing process

During the preparation of this work, the authors used ChatGPT in order to improve readability and language. After using this tool/service, the authors reviewed and edited the content as needed and take full responsibility for the content of the publication.

## Web resources

BafCalculator, https://github.com/BCM-Lupskilab/BafCalculator
Clustalo, http://lilab2.sysu.edu.cn/Tools/msa/clustalo/
dbGaP, https://dbgap.ncbi.nlm.nih.gov/home/
GenBank, https://www.ncbi.nlm.nih.gov/genbank/
gnomAD, https://gnomad.broadinstitute.org/
MCSM-PPI2, https://biosig.lab.uq.edu.au/mcsm_ppi2/
MetaDome, https://stuart.radboudumc.nl/metadome/
OMIM, http://www.omim.org/
RCSB, https://www.rcsb.org/structure/6E7C

## Supplemental information

Document S1. Table S1

Document S2. Article plus supplemental information
