# Supplementary material for: Expanding the clinical and molecular spectrum of TUBB2B through distinct variants identified across multiple families
Source: HGG Adv. 2026 Jul 17;7(4):100654. doi: 10.1016/j.xhgg.2026.100654 (PMC13471236; doi:10.1016/j.xhgg.2026.100654)
Supplement: Supplementary file 1 [file mmc1.pdf]

HGGA, Volume 7

Supplemental information

Expanding the clinical and molecular spectrum of *TUBB2B* through distinct variants identified across multiple families

Shaghayegh T. Beheshti, Angad Jolly, Ahmed K. Saad, Haowei Du, Lauren E. Westerfield, Chloe Munderloh, Divya Kalra, Yifan Wu, Yi Chen, Marie-Claude Gingras, Shalini N. Jhangiani, Sarenur Yilmaz, Maha S. Zaki, Daniel G. Calame, Davut Pehlivan, Richard A. Gibbs, Richard A. Lewis, James R. Lupski, and Jennifer E. Posey

Table S1. Primer sequences used for amplification and Sanger sequencing of *TUBB2B* variants in four affected individuals.

Family ID	Individual ID	Gene	Variant (cDNA:protein)	Forward Primer	Reverse Primer
3	BAB9222	*TUBB2B*	c.43C>A p.(Gln15Lys)	GACACACCCTCGTCTTCTCC	CTAGGGCTCGGAGCATTACG
1	BAB11820	*TUBB2B*	c.292G>A p.(Gly98Arg)	CGTTGTCAATGCAGTAGGTTTC	GTGTCCTGGCCTTCCTTATTTA
4	BAB13277	*TUBB2B*	c.145G>A p.(Val49Ile)	AGGCACTCACCAAACACGAA	CATTGTGAGCCTTGGCGTTT
4	BAB13280	*TUBB2B*	c.145G>A p.(Val49Ile)	AGGCACTCACCAAACACGAA	CATTGTGAGCCTTGGCGTTT
